# Health related quality of life and its determinants in COVID-19 patients

**DOI:** 10.5578/tt.20239706

**Published:** 2023-09-22

**Authors:** S. Satar, M.E. Şahin, P. Ergün

**Affiliations:** 1 Pulmonary Rehabilitation Center, Clinic of Chronic Respiratory Failure, Ankara Atatürk Sanatorium Training and Research Hospital, Ankara, Türkiye

**Keywords:** COVID-19, health-related quality of life, predictors, SF-36

## Abstract

**ABSTRACT:**

Health related quality of life and its determinants in COVID-19 patients

**Introduction:**

One of COVID-19’s limitations is the reduced quality of life (QoL) caused by variety of underlying reasons. Even though multiple papers in the literature reveal a worsening of QoL after COVID-19, there is currently inadequate evidence on which patients’ QoL is impacted the most. Our study’s aim was to determine which patients’ quality of life was most compromised so that interventions for poor QoL should not be overlooked in the post-disease assessments of those in the risk group.

**Materials and Methods:**

Patients referred to our pulmonary rehabilitation center for Long COVID symptoms had their dyspnea perception, body composition, exercise capacity, muscle strengths, and psychological state evaluated. In addition, SF-36 was used to assess their QoL. After obtaining all medical data, the patients were separated into three groups based on whether they had the disease as an outpatient, inpatient in the hospital, or in the intensive care unit. The Anova and Kruskal Wallis tests were utilized in the statistical analysis of demographic data among patient groups. Pearson’s test was used for normal distributions, whereas Spearman’s test was used for non-normal distribution analyses. The factors affecting QoL were investigated using multivariate linear regression analysis.

**Results:**

The majority of 173 study participants had poor QoL. Low exercise capacity (p= 0.026), impaired psychosocial status (p= 0.034 for anxiety, p= 0.022 for depression), and increased fatigue (p= 0.001) were the factors affecting SF-36’s physical component summary (PCS), whereas young age (p= 0.026), male sex (p= 0.037), impaired psychosocial status (p< 0.001 for anxiety, p= 0.002 for depression), and increased fatigue (p= 0.005) were the factors affecting the SF-36’s mental component summary (MCS).

**Conclusion:**

Young age, male sex, reduced exercise capacity, poor psychosocial status, and increased fatigue are predictors for impaired QoL after COVID- 19. Therefore, non-medical treatment options that improve QoL should be considered in the follow-up of these patients.

## Abstract (tr)

**ÖZ:**

Yoğun bakımdaki yaşlı kırılgan hastalarda diyafram ultrasonu mekanik ventilatörden ayrılmayı hızlı yüzeyel solunum indeksine göre daha iyi öngördürebilir mi?

**Giriş:**

COVID-19’un sınırlamalarından biri, altta yatan çeşitli nedenlerden kaynaklanan yaşam kalitesinin düşmesidir. Literatürdeki çok sayıda makale COVID-19’dan sonra yaşam kalitesinin kötüleştiğini ortaya koysa da şu anda hangi hastaların yaşam kalitesinin en çok etkilendiğine dair yeterli kanıt bulunmamaktadır Çalışmamızın amacı, risk grubundaki hastaların hastalık sonrası değerlendirmelerinde yaşam kalitesinin bozulmasına yönelik müdahalelerin göz ardı edilmemesi için hangi hastaların yaşam kalitelerinin en fazla etkilendi- ğini belirlemektir.

**Materyal ve Metod:**

Uzun COVID semptomları nedeniyle pulmoner rehabilitasyon merkezimize başvuran hastaların nefes darlığı algısı, vücut kompozisyonu, egzersiz kapasitesi, kas kuvvetleri ve psikolojik durumları değerlendirildi. Ayrıca SF-36, yaşam kalitesi durumlarını değerlendirmek için kullanıldı. Tüm tıbbi veriler elde edildikten sonra hastalar ayaktan, hastanede yatarak ve yoğun bakımda yatarak tedavi görme durumlarına göre üç gruba ayrıldı. Hasta grupları arasındaki demografik verilerin istatistiksel analizinde Anova ve Kruskal Wallis testlerinden yararlanıldı. Normal dağılım gösteren parametreler için Pearson testi, normal dağılım göstermeyenler içinse Spearman testi kullanıldı. Yaşam kalitesini etkileyen faktörler çok değişkenli lineer regresyon analizi kullanılarak analiz edildi.

**Bulgular:**

Yüz yetmiş üç çalışma katılımcısının çoğunluğu kötü bir yaşam kalitesine sahipti. Düşük egzersiz kapasitesi (p= 0,026), bozulmuş psikososyal durum (anksiyete için p= 0,034, depresyon için p= 0,022) ve artan yorgunluk (p= 0,001) SF-36’nın fiziksel bileşen özetini (PCS) etkileyen faktörlerken; genç yaş (p= 0,026), erkek cinsiyet (p= 0,037), bozulmuş psikososyal durum (anksiyete için p< 0,001, depresyon için p= 0,002) ve artan yorgunluk (p= 0,005) SF-36’nın zihinsel bileşenini (MCS) etkileyen faktörlerdir.

**Sonuç:**

Genç yaş, erkek cinsiyet, azalmış egzersiz kapasitesi, kötü psikososyal durum ve artan yorgunluk COVID-19 sonrası yaşam kalitesinin bozulması için öngörücülerdendir. Bu nedenle bu hastaların takibinde yaşam kalitesini iyileştiren ilaç dışı tedavi seçenekleri de değerlendirilmelidir.

## Introduction

Coronavirus disease-2019 (COVID-19) can manifest as mild disease, pneumonia, severe pneumonia, acute respiratory distress syndrome (ARDS), sepsis, and septic shock, especially in the elderly and those with comorbidities ( 1 , 2 ). In fact, in a report from United States, it has been mentioned that COVID-19 can cause prolonged morbidity, even in young persons who do not have underlying chronic diseases ( 3 ). According to World Health Organization’s (WHO) 2021 report, 10% to 20% of patients experience a range of mid-and long-term sequelae after they recover ( 4 ). This is why people with COVID-19 have a worse quality of life in the early and late stages of the disease ( 5 ). A review has shown that survivors of COVID-19 have poor levels of physical function, activities of daily living, and health-related quality of life (HRQoL) even six months after the infection ( 6 ). Another study on long COVID has found that regardless of the initial disease severity, HRQoL, exercise capacity, and mental health continue to improve throughout two years ( 7 ).

Although the quality of life (QoL) deteriorated after COVID-19 was mentioned in many publications, the factors affecting this have been investigated in very few studies. In one of these reviews, HRQoL was documented in patients with acute COVID, females, elderly people, those with more severe illnesses, and people from low-income countries ( 8 ).

Actually, in the multidisciplinary management of COVID-19, it is essential to understand the psychological impact as well as the physical symptoms in detail and to reveal the reasons for its formation. So in this study, it was aimed to examine the quality of life of 173 patients who survived COVID-19 and to reveal which factors affected their impaired QoL the most.

## MATERIALS and METHODS

### Study Population

**Figure 1:**
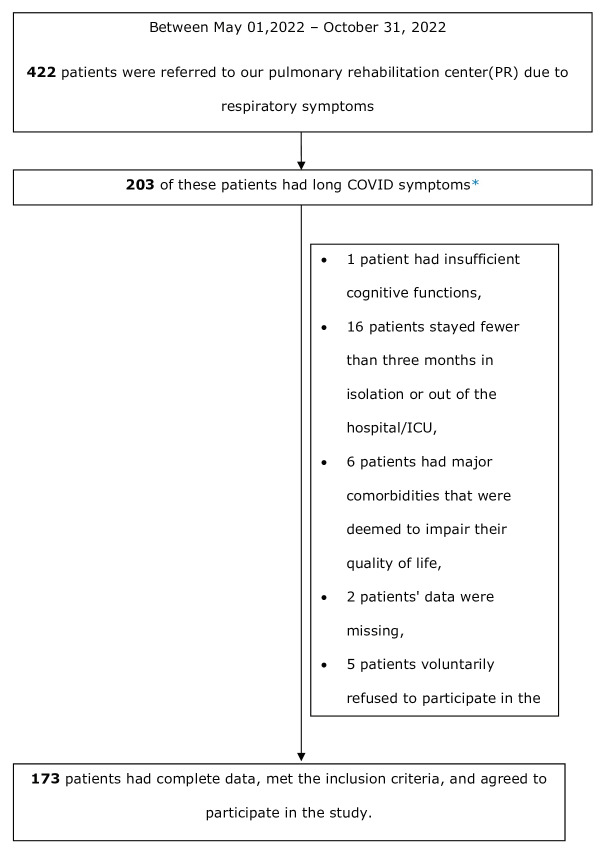
Flow diagram of the study patients ( 3 ).

This single-center, prospective, cross-sectional study included 203 patients who were referred to our pulmonary rehabilitation (PR) center due to long COVID. The mean time of all our patients from the time of their reverse transcription polymerase chain reaction (RT-PCR) positivity for COVID-19 until they applied for PR was 144 days. Of these, 173 patients had complete data, met the inclusion criteria, and agreed to participate in the study ( Figure 1 ). Inclusion criteria for the study involved being above the age of 18, being diagnosed with COVID-19 with positive RT-PCR, and still experiencing prolonged COVID-19 symptoms at least three months after the end of isolation or hospital (ward) or intensive care unit (ICU) discharge. Patients under the age of 18, those with insufficient cognitive functions, those who have been out of isolation or hospital/ICU for less than three months, patients with severe comorbidities thought to impair their quality of life, such as cancer, physical, ortopedic or neurological limitations, patients with incomplete data, and patients who voluntarily refused to participate in the study were all excluded. The patients included in the study were separated into three groups based on whether they had the disease as an outpatient, inpatient in the hospital, or in the intensive care unit. All patients who participated signed an informed consent form.

After submitting an application to the Turkish Ministry of Health’s Scientific Research Platform and receiving approval from it, we applied to the “Medical Specialization Education Board” in our institution and received approval for our study on 26 April 2022 with decision number 2012-KAEK-15/2505.

### Outcome Parameters

At the time of admission to PR, the pulmonologist questioned all patients about their detailed medical history. Then the patients’ postero-anterior (PA) chest radiographs were performed and evaluated by two different pulmonologists, and the amount of involvement was decided by consensus. PA chest radiographs were divided into six regions, one for each lung’s upper, middle, and lower zones, and the number of zones compatible with COVID-19 was recorded. A spirometry was used to measure pulmonary function forced expiratory volume in 1 second (FEV_1_), forced vital capacity (FVC), and FEV_1_/FVC) ( 9 ). The modified medical research council (mMRC) scale was used to measure dyspnea perception ( 10 ). Body compositions were measured by the bioelectrical impedance method. In order to determine exercise capacity, incremental shuttle walking test (ISWT) was utilized ( 11 ). Respiratory muscle strength maximal inspiratory pressure (MIP), maximal expiratory pressure (MEP) was assessed using a respiratory pressure meter (micro-RPM). To examine peripheral muscle strength, hand grip test with hand dynamometer was applied. Health-related quality of life was assessed by using the 36-item short form health survey (SF-36) ( 12 ). The SF-36 version 1.0 is a 36-item short form questionnaire that measures eight components of health-related quality of life: physical functioning (PF), role limitation due to physical problems (RP), bodily pain (BP), general perception of health (GH), energy and vitality (VT), social functioning (SF), role limitation due to emotional problems (RE), and mental health (MH). Furthermore, the results of our patients were calculated in accordance with the study of Demiral et al., in which the population norms of the SF-36 health survey in the Turkish urban population were obtained ( 13 ). Using the usual SF-36 scoring methods, item scores were coded, totaled, and converted into a scale of 0 (worst) to 100 (best) for each quality of life dimension examined. Scores for the physical and mental component summary (PCS and MCS, respectively) were also determined by using oblique scoring algoritms. According to this study, the mean of PCS and MCS was approved as 50, with a standard deviation of 10, with high scores representing good quality of life and low scores representing bad quality of life. Psychological status was determined using hospital anxiety and depression (HAD) scores ( 14 , 15 ).

There are 14 items total on this scale, which has a two-factor structure. Seven of these questions evaluate anxiety, while the remaining evaluate depression. The total score for anxiety and depression ranges from 0 to 21. Normal emotional status is indicated by a score between 0 and 7, and scores greater than 7 were indicative of an anxiety or depressive disorder. The Turkish version of the HAD has demonstrated validity and reliability in both hospitalized patients and healthy college students ( 15 ). Finally, the fatigue severity scale (FSS), for which the Turkish version was also validated, was used to evaluate fatigue levels ( 16 ). The FSS has nine questions that assess the severity of fatigue symptoms throughout the previous week. A score of four or above indicates extreme exhaustion.

### Statistical Analysis

IBM SPSS version 26.0 statistical analysis soft ware was used (IBM SPSS Statistics for Windows, Chicago, IL, USA). While categorical variables were expressed as number and percentages (%), numerical variables were expressed as mean and standard deviation. Visual (histograms, probability plots) and analytical (Shapiro-Wilk, Skewness, and Kurtosis tests) techniques were used to check the variables’ normality. Anova and Kruskal Wallis tests were used for normal and non-normal quantitative variables, respectively, in the statistical analysis of demographic data among the groups created based on hospitalization status (outpatient, hospital, or ICU). For post-hoc analyses, Tukey HSD and Games-Howell tests were employed. Using Pearson’s test, the correlation between two numerical variables with normal distribution and a linear correlation was examined. Spearman’s test was used to assess the correlation between variables that did not exhibit a normal distribution. Multivariate linear regression analysis was used to analyze the factors that affected the quality of life measured by SF-36. Statistical significance was determined using a 0.05 p-value.

## RESULTS

**Figure 2:**
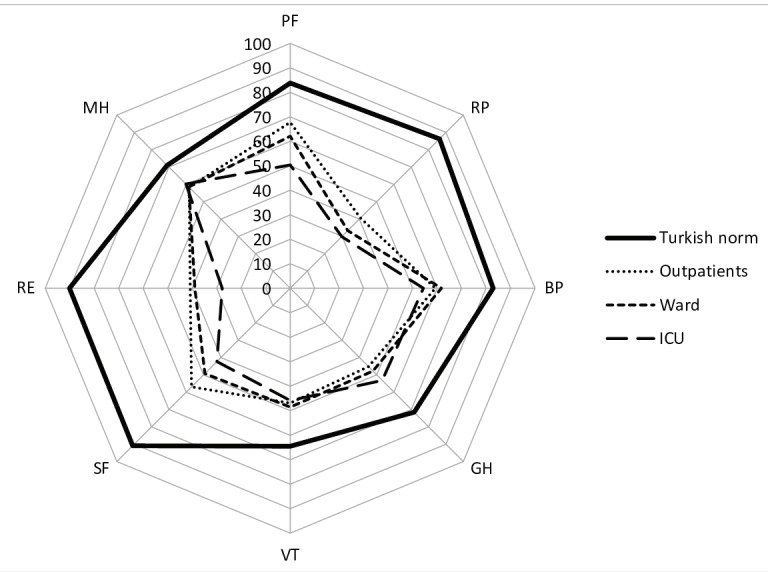
The results of the SF-36 component scores and the normal valus of the Turkish population in the groups formed according to the way of experiencing the disease (outpatient, hospitalized in ward, or ICU) ( 13 ). PF: Physical functioning, SF: Social functioning, RP: Role limitation due to physical problems, RE: Role limitation due to emotianal problems, MH: Mental health, VT: Energy and vitality, BP: Bodily pain, GH: General perception of health, ICU: Intensiva care unit. *There were statistically significant differences only in the PF and SF components between the patient groups (p= 0.013, and p= 0.042, recpectively).

**Figure 3:**
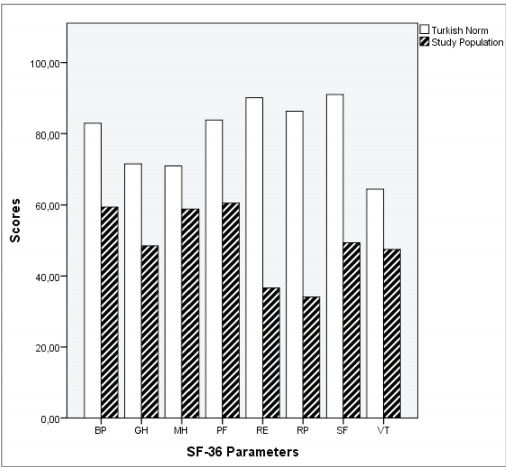
Mean scores in SF-36 for COVID-19 patients vs. Turkish population norms ( 13 ). BP: Bodily pain, GH: General perception of health, MH: Mental health, PF: Physical functioning, RE: Role limitation due to emotional problems, RP: Role limitation due to physical problems, SF: Social functioning, VT: Energy and vitality. *There was a statistically significant difference in all components (p< 0.01).

**Figure 4:**
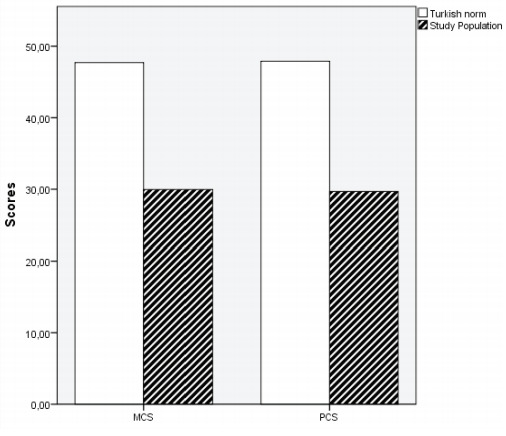
Mean scores in the mental and physical component summary (MCS and PCS, respectively) for COVID-19 patients vs. Turkish population norms ( 13 ). MCS: Mental component summary, PCS: Physical component summary. *There was a statistically significant difference in both components summaries (p< 0.01).

Among our patients, 119 (68.8%) were males. Between the outpatient, ward, and ICU groups, there was a significant sex difference (p= 0.001). Compared to 104 patients (60.1%), only 69 of our patients (39.9%) were receiving long-term oxygen treatment (LTOT) at home. Fifty-three patients (30.6%) had no other disease, and 120 patients (69.4%) had comorbidities. When we categorized our patients based on how the disease progressed, we discovered that 40 (23.1%) recovered at home, 91 (52.6%) were hospitalized, and 42 (24.3%) were treated in the ICU. Figure 2 shows the results of the SF-36 component scores of the patient groups formed based on the mode of disease experience (outpatient, ward, intensive care) and the normal values of the Turkish population. There were statistically significant differences between the patient groups only in the PF and SF components (p= 0.013 and p= 0.042, respectively), and all components of the SF-36 were worse than the Turkish population norms. Figure 3 and Figure 4 provide a comparison of eight sub-parameters of SF-36s among all patients, as well as PCS and MCS data from the Turkish population, respectively. When the eight sub-parameters of the SF-36 and the PCS and MCS scores of all patients without grouping were compared with the Turkish population norms, there was a statistically significant difference in all parameters (p< 0.001).

In addition, there was a statistically significant difference between the three groups in terms of sex (p= 0.001), smoking history (p= 0.002), LTOT (p< 0.001), mMRC (p< 0.001), FVC (p< 0.001), number of lung zones involved in chest radiographs (p< 0.001), ISWT (p< 0.001), MIP (p= 0.034), MEP (p= 0.031), hand grip strength (p= 0.043), PF (p= 0.013), and SF (p= 0.042). Details of the parameters are given in Table 1 .

In the SF-36 correlation analysis, mMRC and ISWT were found to be significantly correlated with all parameters of SF-36 except BP (p= 0.384) and MH (p= 0.099). Furthermore, HAD scores and FSS were shown to be correlated to all SF-36 components. While the number of days spent in the hospital was correlated to both PF (p< 0.001) and PCS (p= 0.042), the number of days spent in the intensive care was solely related to PF (p= 0.004). There was no correlation between body mass index (BMI) and any of the SF-36 components. Table 2 details the correlations.

In the regression analysis, age, sex, smoking history, LTOT, mMRC, length of stay in hospital, FEV_1_, ISWT, HG strength, HAD, and FSS were included. Although mMRC, FEV_1_, and use of LTOT were correlated with both PCS and MCS, regression analysis showed no impact on quality of life. And while the factors affecting PCS were ISWT (p= 0.026), HAD (p= 0.034 for anxiety, p= 0.022 for depression), and FSS (p= 0.001), the factors affecting MCS were determined as age (p= 0.026), gender (p= 0.037), HAD (p< 0.001 for anxiety, p= 0.002 for depression), and FSS (p= 0.005). Details of the multivariate linear regression analysis are shown in Table 3 .

**Table 1:** Summary of the parameters of patients in general, as well as those with a history of outpatient, hospitalization in ward, or intensive care unit hospitalization

		All Patients (n= 173)	Outpatient (n= 40)	Ward (n= 91)	ICU (n= 42)	p
Age		56.21 ± 10.64	54.45 ± 9.80	56.49 ± 11.59	57.26 ± 9.20	0.45
Sex m/f n (%)		119 (68.8%)/54 (31.2%)	18 (45%)/22 (55%)	71 (78%)/20 (22%)	30 (71.4%)/12 (28.6%)	0.001
Smoking (pack year)		28.22 ± 21.22	25.25 ± 17.72	32.53 ± 21.11	17.66 ± 21.90	0.002
Comorbidity n (%) (-/+)		53 (30.6%)/120 (69.4%)	8 (20%)/32 (80%)	29 (31.9%)/62 (68.1%)	16 (38.1%)/26 (61.9%)	0.195
LTOT n(%) (-/+)		104 (60.1%)/69 (39.9%)	36 (90%)/4 (10%)	55 (60.4%)/36 (39.6%)	13 (31%)/29 (69%)	<0.001
mMRC		2.22 ± 0.97	1.70 ± 0.79	2.21 ± 0.089	2.75 ± 1.03	<0.001
n hospital days		-	0	12.52 ± 9.17	32.78 ± 15.47	
n_ICU days_		-	0	0	15.62 ± 10.63	
X-ray (zones)		2.51 ± 1.99	1.63 ± 2.047	2.50 ± 1.83	3.41 ± 1.93	<0.001
FVC		85.67 ± 22.49	93.74 ± 20.50	86.13 ± 22.77	73.97 ± 19.74	<0.001
FEV_1_		82.93 ± 22.13	87.64 ± 21.03	82.57 ± 23.48	77.73 ± 19.03	0.179
FEV_1_/FVC		78.65 ± 13.31	77.04 ± 11.09	77.48 ± 13.00	83.67 ± 15.66	0.060
BMI		29.06 ± 5.23	28.73 ± 4.05	28.73 ± 5.56	30.12 ± 5.52	0.343
ISWT		345.67 ± 139.65	387.75 ± 87.07	360.58 ± 150.49	267.63 ± 131.17	<0.001
MIP		95.31 ± 29.25	82.89 ± 26.13	98.84 ± 25.36	99.88 ± 35.52	0.034
MEP		122.44 ± 35.40	107.96 ± 28.55	129.68 ± 33.08	123.90 ± 41.47	0.031
HGS		30.90 ± 10.60	28.59 ± 10.75	32.92 ± 10.23	28.79 ± 10.64	0.043
HADa		7.20 ± 3.87	7.85 ± 3.88	6.90 ± 3.91	7.22 ± 3.81	0.438
HADd		6.83 ± 3.41	6.70 ± 3.30	6.92 ± 3.52	6.76 ± 3.33	0.932
SF-36	PF	60.55 ± 27.82	67.75 ± 22.18	62.09 ± 26.82	50.36 ± 32.20	0.013
	RP	34.10 ± 38.50	40.63 ± 38.26	33.24 ± 38.74	29.76 ± 38.34	0.424
	BP	59.42 ± 27.75	59.31 ± 28.76	61.78 ± 25.60	54.40 ± 31.10	0.364
	GH	48.53 ± 19.52	45.25 ± 15.27	47.80 ± 20.51	53.21 ± 20.47	0.160
	VT	47.49 ± 19.07	46.88 ± 18.56	48.52 ± 20.00	45.83 ± 17.73	0.735
	SF	49.36 ± 26.59	57.00 ± 25.88	49.28 ± 26.24	42.26 ± 26.61	0.042
	RE	36.66 ± 41.67	40.83 ± 43.68	38.93 ± 40.96	27.77 ± 40.93	0.277
	MH	58.87 ± 18.81	58.28 ± 18.11	58.52 ± 19.86	60.19 ± 17.46	0.873
	PCS	29.69 ± 12.61	31.47 ± 11.93	30.07 ± 12.42	27.15 ± 13.55	0.277
	MCS	29.96 ± 14.51	30.69 ± 14.77	30.28 ± 15.08	28.55 ± 13.19	0.765
FSS		4.51 ± 1.78	4.57 ± 1.83	4.48 ± 1.76	4.50 ± 1.80	0.970

ICU: Intensive care unit, n: Number, m/f: Male/female, (-/+): Absent/available, LTOT: Long term oxygen treatment, mMRC: Modified medical research council, n_hospital days_: Number of days in hospital, n_ICU days_: Number of days in ıntensive care unit, FVC: Forced vital capacity, FEV_1_ : Forced expiratory volume in one second, BMI: Body mass index, FFMI: Fat-free mass index, ISWT: Incremental shuttle walking test, MIP: Maximal inspiratory pressure, MEP: Maximal expiratory pressure, HGS: Hand grip strength, HADa: Hospital anxiety score, HADd: Hospital depression score, SF-36: Short form-36, PF: Physical functioning, RP: Role limitation due to physical problems, BP: Bodily pain, GH: General perception of health, VT: Energy and vitality, SF: Social functioning, RE: Role limitation due to emotional problems, MH: Mental health, PCS: Physical component summary, MCS: Mental component summary, FSS: Fatigue severity score.

**Table 2:** Correlation analysis

		PF	RP	BP	GH	VT	SF	RE	MH	PCS	MCS
Age	r	-0.024	-0.010	0.175	0.155	0.117	0.009	0.016	0.140	0.082	0.119
	p	0.757	0.896	0.022	0.041	0.125	0.910	0.833	0.067	0.283	0.119
Sex	r	-0.007	-0.022	-0.121	-0.173	0.078	0.046	-0.081	-0.017	-0.083	-0.065
	p	0.931	0.777	0.112	0.023	0.306	0.552	0.289	0.826	0.280	0.396
Smoking	r	0.062	0.206	0.122	0.001	0.085	0.092	0.201	0.038	0.163	0.129
	p	0.419	0.007	0.112	0.985	0.267	0.228	0.008	0.620	0.033	0.092
LTOT	r	0.207	0.159	-0.064	0.119	0.113	0.267	0.182	0.046	0.184	0.167
	p	0.007	0.039	0.409	0.123	0.143	<0.001	0.018	0.552	0.017	0.030
mMRC	r	-0.343	-0.248	-0.067	-0.211	-0.278	-0.373	-0.206	-0.127	-0.342	-0.291
	p	<0.001	0.001	0.384	0.006	<0.001	<0.001	0.007	0.099	<0.001	<0.001
n_hospital days_	r	-0.346	-0.188	-0.063	0.040	-0.061	-0.139	-0.108	0.091	-0.199	-0.036
	p	<0.001	0.056	0.525	0.684	0.539	0.160	0.275	0.358	0.042	0.718
n_ICU days_	r	-0.215	-0.029	-0.027	0.093	-0.016	-0.112	-0.044	0.036	-0.074	-0.015
	p	0.004	0.702	0.720	0.223	0.835	0.144	0.569	0.638	0.332	0.844
X- ray (zones)	r	-0.247	-0.165	0.058	0.082	-0.009	-0.289	-0.175	0.101	-0.152	-0.062
	p	0.001	0.031	0.452	0.285	0.908	<0.001	0.022	0.188	0.047	0.421
FVC	r	0.143	0.059	-0.070	0.028	0.006	0.212	0.164	0.038	0.085	0.105
	p	0.084	0.476	0.395	0.732	0.942	0.010	0.047	0.648	0.303	0.205
FEV_1_	r	0.174	0.095	-0.010	0.151	0.100	0.244	0.130	0.090	0.162	0.163
	p	0.034	0.249	0.907	0.068	0.227	0.003	0.116	0.278	0.050	0.048
BMI	r	0.009	0.072	0.030	-0.079	0.036	-0.034	-0.020	-0.121	0.025	-0.055
	p	0.911	0.358	0.700	0.309	0.645	0.665	0.802	0.119	0.751	0.481
ISWT	r	0.317	0.182	0.056	0.165	0.170	0.314	0.214	0.056	0.276	0.216
	p	<0.001	0.020	0.480	0.034	0.030	<0.001	0.006	0.473	<0.001	0.006
MIP	r	0.051	0.056	0.144	0.203	0.222	0.110	0.110	0.050	0.168	0.161
	p	0.583	0.551	0.122	0.028	0.016	0.238	0.236	0.589	0.070	0.083
MEP	r	0.096	0.113	0.134	0.165	0.151	0.094	0.189	<0.001	0.183	0.136
	p	0.324	0.244	0.166	0.088	0.118	0.333	0.050	0.997	0.058	0.161
HGS	r	0.143	0.114	0.064	0.185	0.200	0.100	0.165	0.108	0.178	0.189
	p	0.073	0.157	0.424	0.020	0.012	0.214	0.040	0.180	0.025	0.018
HADa	r	-0.225	-0.250	-0.330	-0.494	-0.484	-0.318	-0.297	-0.570	-0.449	-0.587
	p	0.003	0.001	<0.001	<0.001	<0.001	<0.001	<0.001	<0.001	<0.001	<0.001
HADd	r	-0.264	-0.202	-0.379	-0.395	-0.506	-0.364	-0.201	-0.514	-0.442	-0.543
	p	<0.001	0.008	<0.001	<0.001	<0.001	<0.001	0.009	<0.001	<0.001	<0.001
FSS	r	-0.392	-0.214	-0.255	-0.419	-0.437	-0.299	-0.299	-0.296	-0.433	-0.439
	p	<0.001	0.006	0.001	<0.001	<0.001	<0.001	<0.001	<0.001	<0.001	<0.001

PF: Physical functioning, SF: Social functioning, RP: Role limitation due to physical problems, RE: Role limitation due to emotional problems, MH: Mental health, VT: Energy and vitality, BP: Bodily pain, GH: General perception of health, PCS: Physical component summary, MCS: Mental component summary, mMRC: Modified medical research council, n_hospital days_: Number of days in hospital, n_ICU days_: Number of days in intensive care unit, FVC: Forced vital capacity, FEV_1_: Forced expiratory volume in one second, BMI: Body mass index, ISWT: Incremental shuttle walking test, MIP: Maximal inspiratory pressure, MEP: Maximal expiratory pressure, HGS: Hand grip strength, HADa: Hospital anxiety score, HADd: Hospital depression score, FSS: Fatigue severity score.

**Table 3:** Regression analysis for PCS and MCS

	PCS					MCS				
	Unstandardized Coefficients B	Standardized Coefficients β	t	Sig.	95% CI for B	Unstandardized Coefficients B	Standardized Coefficients β	t	Sig.	95% CI for B
Age	0.207	0.172	1.925	0.057	-0.006/0.419	0.256	0.179	2.252	0.026	0.031/0.480
Sex	1.021	0.037	0.362	0.718	-4.571/6.614	6.309	0.194	2.112	0.037	0.392/12.227
Smoking	0.028	0.043	0.544	0.587	-0.075/0.131	0.055	0.071	0.996	0.321	-0.054/0.164
LTOT	-0.930	-0.036	-0.418	0.677	-5.338/3.478	-0.753	-0.024	-0.320	0.750	-5.417/3.911
mMRC	-1.203	-0.085	-0.861	0.391	-3.970/1.564	-1.155	-0.069	-0.781	0.436	-4.083/1.772
n_hospital days_	-0.022	-0.025	-0.300	0.765	-0.167/0.123	0.063	0.059	0.812	0.418	-0.090/0.216
FEV_1_	0.005	0.008	0.087	0.931	-0.100/0.109	-0.001	-0.002	-0.025	0.980	-0.112/0.110
ISWT	0.025	0258	2.251	0.026	0.003/0.047	0.022	0.189	1.852	0.067	-0.002/0.045
HGS	-0.089	-0.070	-0.685	0.495	-0.347/0.168	0.071	0.046	0.513	0.609	-0.202/0.343
HADa	-0.647	-0.196	-2.143	0.034	-1.245/-0.049	-1.448	-0.369	-4.535	0.000	-2.081/-0.816
HADd	-0.815	-0.209	-2.313	0.022	-1.512/-0.117	-1.154	-0.249	-3.096	0.002	-1.892/-0.416
FSS	-1.948	-0.273	-3.454	0.001	-3.064/-0.831	-1.701	-0.201	-2.852	0.005	-2.883/-0.520

CI: Confidence interval, PCS: Physical component summary, MCS: Mental component summary, LTOT: Long term oxygen treatment, mMRC: Modified medical research council, n_hospital days_: Number of days in hospital, FEV_1_: Forced expiratory volume in one second, ISWT: Incremental shuttle walking test, HGS: Hand grip strength, HADa: Hospital anxiety score, HADd: Hospital depression score, FSS: Fatigue severity score.

## DISCUSSION

Based on our study results, the quality of life for most of our study participants was deteriorated. Our findings also show that regardless of the COVID-19 severity, age, sex, exercise capacity, psychosocial status, and fatigue can all be considered to be factors affecting a person’s quality of life. Low ISWT was found to be a predictor for poor PCS, while young age and male sex were determinants for poor MCS. Also, we discovered that increased fatigue and a poor psychosocial status are predictors for both PCS and MCS.

In our study, we evaluated our patients at the earliest three months after COVID-19 and divided them into three groups as outpatient, hospitalized and ICU patients. Among these three groups, only PF and SF were significantly better in the outpatients compared to the other groups, while there was no difference between the groups in terms of PCS and MCS, and these values were also very poor in outpatients. Similar to our findings, in a study evaluating the general and respiratory-specific QoL in COVID-19 patients who were never hospitalized, it has been discovered that three months after the onset of symptoms, both generic and respiratory-specific QoL are negatively impacted in these patients ( 17 ). Also, when the HRQoL scores are compared to population norms in a population-based cohort study of non- hospitalized participants four months after their COVID-19, all parameters except PF and BP show a statistically significant decline ( 18 ). These outcomes confirm that regardless of the severity of the disease, the quality of life for all COVID-19 patients may deteriorate.

Moreover, a study that looked at the QoL of COVID- 19 patients hospitalized in the ICU has divided the patients into three groups: those receiving high-flow nasal oxygen, those under non-invasive mechanical ventilator support, and those under invasive mechanical ventilator support. It has been discovered that there was no difference in HRQoL between the groups. The only difference between the groups’ results after adjusting for age, sex, BMI, and comorbidities was that patients receiving invasive ventilation experienced more severe bodily pain ( 19 ). Approximately 24% of the patients in our study were admitted to the ICU, but no clear information on its procedures was available. According to our results, ICU admission did not predict PCS or MCS and was only significantly correlated with PF.

Thus, the findings of our study as well as those of other studies in the literature demonstrate that, apart from the COVID-19 pandemic’s severe morbidity and mortality, it also negatively affects people’s QoL for a variety of reasons, including social isolation, limitations on social communication, a decrease in physical activity, and financial difficulties. As a result, poor QoL takes up a significant portion of prolonged COVID-19 findings, and the number of studies on this topic is growing. A review about QoL during acute and long COVID-19 periods has discovered that patients with acute COVID had lower HRQoL scores than patients with long COVID ( 8 ). While the MCS in the acute period was marginally higher than the PCS, long COVID revealed the opposite ( 8 ). This illustrates that, while the disease’s physical symptoms get better over time, its psychological effects continue to deteriorate. According to the results of another review investigating impaired QoL and related factors after COVID-19, regardless of the time following discharge or recovery, female sex, older age, comorbidities, ICU admission, prolonged ICU stay, and being mechanically ventilated are the factors most associated with impaired QoL ( 20 ). In this review, 7 of the 21 studies used the SF-36 to assess HRQoL, and they discovered that RP and PF were the most and least affected parameters, respectively. Similar to this review, in our study, RP was the most affected parameter with the lowest scores, while PF was the parameter with the highest score and the best condition in terms of quality of life. However, in contrast to the findings of this review, we discovered that women and older age were predictors of good quality of life, particularly the better mental component. This is likely because women tend to have more outpatient or milder conditions than men. Additionally, despite having a strong correlation with PF and PCS in our study, regression analysis revealed that the number of hospital days had no effect on QoL. Also, LTOT use after discharge was linked to PF, RP, SF, RE, PCS, and MCS in the correlation analysis but had no significant impact on QoL in the regression analysis.

In a study investigating the factors affecting QoL one month after discharge in hospitalized patients due to COVID-19, smoking was related to RE but was not a predictor of PCS or MCS ( 21 ). A further result wast that, unlike MCS, PCS is more likely to develop in people who are overweight or obese ( 21 ). Contrarily, in our study, correlation analysis with cigarette pack year revealed a relation between RP, RE, and PCS, but no impact on PCS or MCS in regression analysis. Furthermore, despite the fact that all of our study participants were overweight, there was no correlation between BMI and any SF-36 parameter.

COVID-19 causes pathological processes in the respiratory, cardiovascular, and musculoskeletal systems as a result of systemic inflammation, leading to impaired function and decreased exercise capacity ( 22 ). Although it is anticipated that exercise capacity will return to normal as the disease’s inflammation lessens over time, post hoc analysis of a study done at the 5th month after acute illness revealed that there were no differences between community-recovered and healthy control groups in any cardiopulmonary exercise testing or spirometry value, but the hospitalized-recovered group and healthy controls, however, differed from one another ( 23 ). According to another study on impaired pulmonary function, exercise capacity, and health status following COVID-19, there were significant positive correlations between lung function parameters and a number of SF-36 domains (PF, RP, GH, SF, and RE). Additionally, all of the SF-36 domains and 6-minute walking distance (6MWD) had statistically significant positive correlations ( 24 ). On the contrary, in a prospective longitudinal study, the impact of COVID-19 on physical functions was examined at 10-weeks, six-months, and the first year following discharge, and it was found that although almost half of the patients’ exercise capacity reached the pre-COVID-19 period, there was no significant difference in mean scores of the eight SF-36 domains over the one-year period ( 25 ). In our study, which was carried out about 144 days after an acute illness, there was no healthy control group but there were significant differences between the outpatient group, hospitalized patients, and ICU inpatients in terms of exercise capacity measured by ISWT. And the results of our regression analysis showed that ISWT was only a predictor of PCS but not MCS, despite the fact that it was correlated with all SF-36 domains apart from BP and MH.

Long COVID includes psychological findings as well as physical findings, and these, like others, impair quality of life in the long term. Looking at the results of a systematic review and meta-analysis examining 151 studies, participants with a higher risk of long-term sequelae are older, mostly male, living in high-income countries and having a more severe status in acute infection. In addition, survivors with mild infections have a higher incidence of anxiety and memory problems, even at least 12 months after recovery ( 26 ). The study by Albu et al. ( 27 ) in post-COVID-19 patients with sequelae and persistent symptoms, who were enrolled in the outpatient rehabilitation program, has demonstrated a significant association between poor HRQoL and fatigue and anxiety/depression. In a study from another PR center, patients’ mean total QoL and its dimensions-including general health, physical status, emotional status, and social function-have significantly improved following a two-week exercise-based PR intervention ( 28 ). According to this study’s findings, PR might be useful for improving the oxygen saturation, lowering dyspnea and pulse rate, and improving the QoL of patients with severe COVID-19 after discharge from ICU. Also, in another study with 102 non-hospitalized COVID-19 patients, chest pain, dyspnea, anxiety or depression, post-traumatic stres disorder (PTSD), and fatigue/muscle weakness have been found to be risk factors for impaired HRQoL ( 29 ). In line with the findings of all these studies, fatigue, anxiety, and depressive symptoms were found to be correlated with each SF-36 domain in our study, and high FSS and HAD scores were revealed to be indicators of poorer quality of life.

The strengths of our study are that besides being a methodologically prospective study, the QoL of patients was evaluated with the SF-36, which is an objective test and provides information about different conditions from many sub-domains. Similar to this, the techniques (ISWT, HG strength, body composition, etc.) applied by the professional multidisciplinary team provided incredibly detailed and comprehensive information about the patients. Our study does have some limitations, though. Firstly, since this is a single-center study, few patients are taken into account. Also, the long-term effects of medications on QoL could not be included in our statistical analyses because the patients did not provide detailed information about their treatments used during hospitalization. Finally, a larger sample size would have likely increased the generalizability of the results.

## CONCLUSION

Even three months after the disease, one of the post-COVID-19 limitations is that the decline in quality of life persists. Health care providers should assess and manage patients in accordance with their individual needs when developing strategies to improve the QoL of post-COVID-19 patients during follow-up visits. This is especially relevant for young, male patients who have low exercise capacity, impaired psychosocial status, and increased fatigue. The use of non-medical treatment modalities like pulmonary rehabilitation, which have been shown to improve quality of life, should be encouraged.

## Ethical Committee Approval

This study wass approved by Ankara Atatürk Sanatorium Training and Research Hospital Clinical Research Ethics Committee (Decision no: 2012-KAEK-15/2505, Date: 26/04/2022).

## CONFLICT of INTEREST

The authors declare no relevant financial disclosures or conflicts of interest. This research received no specific grant from any funding agency in the public, commercial, or not-for-profit sectors.

## AUTHORSHIP CONTRIBUTIONS

Concept/Design: All of authors

Analysis/Interpretation: SS, MEŞ

Data acqusition: SS, MEŞ

Writing: All of authors

Clinical Revision: All of authors

Final Approval: All of authors
